# An Empirical Model to Predict the Diabetic Positive Using Stacked Ensemble Approach

**DOI:** 10.3389/fpubh.2021.792124

**Published:** 2022-01-21

**Authors:** Sivashankari R., Sudha M., Mohammad Kamrul Hasan, Rashid A. Saeed, Suliman A. Alsuhibany, Sayed Abdel-Khalek

**Affiliations:** ^1^School of Information Technology and Engineering, Vellore Institute of Technology (VIT), Vellore, India; ^2^Center for Cyber Security, Faculty of Information Science and Technology, Universiti Kebangsaan Malaysia, Bangi, Malaysia; ^3^Department of Computer Engineering, College of Computers and Information Technology, Taif University, Taif, Saudi Arabia; ^4^Department of Computer Science, College of Computer, Qassim University, Buraydah, Saudi Arabia; ^5^Mathematics and Statistics Department, College of Science, Taif University, Taif, Saudi Arabia; ^6^Mathematics Department, Sohag University, Sohag, Egypt

**Keywords:** random forest, KNN classifier, decision tree, gradient boosting, SVM and Gaussian Naïve Bayes, PIMA dataset, healthcare systems

## Abstract

Today, disease detection automation is widespread in healthcare systems. The diabetic disease is a significant problem that has spread widely all over the world. It is a genetic disease that causes trouble for human life throughout the lifespan. Every year the number of people with diabetes rises by millions, and this affects children too. The disease identification involves manual checking so far, and automation is a current trend in the medical field. Existing methods use a single algorithm for the prediction of diabetes. For complex problems, a single model is not enough because it may not be suitable for the input data or the parameters used in the approach. To solve complex problems, multiple algorithms are used. These multiple algorithms follow a homogeneous model or heterogeneous model. The homogeneous model means the same algorithm, but the model has been used multiple times. In the heterogeneous model, different algorithms are used. This paper adopts a heterogeneous ensemble model called the stacked ensemble model to predict whether a person has diabetes positively or negatively. This stacked ensemble model is advantageous in the prediction. Compared to other existing models such as logistic regression Naïve Bayes (72), (74.4), and LDA (81%), the proposed stacked ensemble model has achieved 93.1% accuracy in predicting blood sugar disease.

## Introduction

People's regular foods contain a vast amount of carbohydrates and calories. Three primary reasons that a person may suffer from diabetes are genetics, lifestyle, and environment. The first reason for diabetic positivity is genetics. Family studies proved that the children whose parents are type 2 [Muoio and Newgard (1)] diabetic have three times more chances to develop diabetic positive than the parents who don't have diabetic positive. Lifestyle is the second reason for the diabetic positive because proven studies show that the individual lifestyle causes diabetic positive even though their ancestors are not diabetic positive. The third reason for the diabetic positive is adopting intricate weight loss mechanisms. It causes kidney failure or heart issues that lead to diabetes positive in the future.

The symptoms for the diabetic positive are eye powerlessness, sudden weight loss, frequent urination, frequent hunger, and thirst. Due to these types of factors, diabetes (sugar patients) risk has increased worldwide. The diabetic disease has become a significant issue in the world. This disease is categorized into two types: type 1 and type 2. According to the International Diabetes Federation (IDF), 463 million people worldwide will have diabetes in 2019 and by 2045, this will rise to 700 million. Early detection of diabetic positives helps reduce patients' medical expenditure, death rate, and risk because they may not have proper health care facilities nearby.

The diabetic patient is categorized into two categories, namely type 1 and type 2. Type 1 diabetic patients are dependent on insulin to control the disease. Type 2 diabetic patients are non-dependent on insulin to control the disease. The diabetic-positive patient has a high risk of several problems such as cardiac arrest, kidney failure, dry skin, paralysis, eye problems, etc. Rural area people are unaware of early-stage symptoms to prevent this diabetic disease, and they are unaware of early-stage symptoms to prevent this diabetic disease.

Diabetes patients increase irrespective of age across all regions of the world, and there is no medicine (vaccine) to prevent it. The diabetic positive type 2 patient body makes gradually used insulin. The insulin increases the blood sugar to save energy into the cells for later usage. The diabetic positive diagnosis or confirmation is made at the hospital through conducting various lab/clinical tests. In this modern life, people are interested in saving their time to save money. It leads to many health complications; one of these is diabetic disease. The proposed system is one of the automated processes of early prediction of diabetic positives. There are several machine learning models were proposed for predicting the person with diabetes positively. The proposed model has outperformed in terms of prediction of diabetic positive compared to other existing models and has achieved 93% accuracy as a detection rate.

### Limitations on Existing Works

The existing approaches in the prediction of diabetic positive are discussed in the next section. Most of the existing works use only a single algorithm, which is used to predict whether the patient is diabetic positive. There are two problems if one algorithm is used for predicting the output. The first problem is that a single algorithm is not sufficient for prediction. Also, the selected dataset may not fit that algorithm. These problems lead to less accuracy in output prediction. The proposed system has used multiple machine-learning algorithms to predict whether the patient has a diabetic positive or not.

### Main Contributions of the Current Work

The proposed system has considered the increasing number of diabetic-positive patients, one of the common problems of all countries globally. Every country has suffered from two main problems without solutions. One is climate change and increasing diabetic positive patients. Thus this paper has investigated the common health issue, diabetic positive, which has no proper software system to predict with high accuracy. This paper has adopted several machine learning algorithms for automating the prediction of diabetic positives. Since the proposed system is a generic model for diabetic prediction, this software system can be used in any region in the world.

## Literature Review

Dhomse Kanchan and Mahale Kishor (2) used multiple machine learning algorithms for rare disease prediction. Kavakiotis et al. (3) proposed multiple machine learning models for the diabetic positive prediction. Kononenko (4) surveyed various medical diagnoses using several artificial intelligent approaches. Kandhasamy and Balamurali (5) used various data mining models such as J48, KNN, and Random Forest, SVM, to predict diabetes mellitus under two different situations (one is before pre-processing and another is after pre-processing). Iyer et al. (6) employed two techniques, namely J48 and Naïve Bayes, to classify diabetic patients. The model J48 approach has achieved 74.87% and Naïve Bayes algorithm has obtained 76.96% accuracy in analyzing diabetes. Ashiquzzaman et al. (7) proposed a Deep Neural Network (DNN) to predict the diabetic positive. The DNN model is also adopted to reduce data overfitting.

Yuvaraj and SriPreethaa (8) adopted Hadoop clustering model for prediction of diabetic positive on big data. Sisodia and Sisodia (9) used Decision Tree, SVM, and Naive Bayes to predict diabetes. The SVM has obtained 65.10% of accuracy using SVM, and Naïve Bayes classifier has obtained 73.82%. Negi and Jaiswal (10) developed a machine learning model for diabetic prediction on different global datasets. This approach is a first attempt of diabetic prediction on global datasets. Soltani and Jafarian (11) proposed a Probabilistic Neural Network (PNN) model for diagnosing diabetes type 2 using the PIMA Indians Diabetes data set. This PNN approach has achieved 90% accuracy in analyzing diabetes. Rakshit et al. (12) used a Two-Class Neural Network to predict diabetes. This model has achieved an 83.3% detection rate of type 2 diabetes. Mamuda and Sathasivam (13) compared four machine learning approaches, Naïve Bayes, Quadratic Discriminant Analysis, Linear Discriminant Analysis, and Gaussian Process Classifier, and obtained the accuracy as 81.97% with respect to cross validation of 10.

Farran et al. (14) proposed several prediction models to predict the risk factor of diabetic two positive patients. Anand and Shakti (15) combined multiple machine learning models to predict the diabetic based on personal lifestyle indicators. Malik et al. (16) proposed a non-invasive detection model for blood glucose level using saliva. Mirshahvalad and Zanjani (17) proposed multiple ensemble techniques for diabetes prediction. Mohebbi et al. (18) developed a deep learning model to detect type 2 diabetics. Pham et al. (19) developed a deep learning model for analyzing medical records to predict the trajectories. Askarzadeh and Rezazadeh (20) proposed a neural network model to achieve an effective training novel optimization algorithm for the clinical data analysis. Rao et al. (21) developed a combined classifiers for disease diagnosis. Kopitar et al. (22) employed three techniques, namely Random Forest algorithm, Naïve Bayes classifier, and KNN, for predicting the diabetic. Apart from these machine learning algorithms for predicting diabetic positive, they also applied XGBOOST, Glmnet, and LightGBM methods for diabetic prediction. Among these methods, the XGBOOST outperformed in diabetic prediction. It has obtained 88% accuracy. Naveen et al. (23) adopted five different machine learning algorithms, SVM, selection Tree, Naive Bayes, Logistic Regression, and KNN, to predict the diabetic positive in the PIMA dataset. This combined machine learning algorithm has obtained 75% accuracy in diabetic prediction.

Butt et al. (24) adopted LSTM to predict the diabetic positive in the PIMA dataset. The machine learning models played a major role in data analysis particularly in clinical data analysis. Thus, the proposed work has adopted machine leaning models for the diabetic positive prediction. Apart from this diabetic positive prediction, the machine learning models are also helpful in other clinical data analysis such as heart disease, cancer tumor, and COVID-19 predictions. The following recent research is evidence for the above statement. Jain et al. (25) proposed several machine learning models to predict COVID-19 positive from B-cell dataset. Shubham et al. (26) proposed deep learning based for identification of glomeruli in the human kidney. Mohan et al. (27) employed two techniques, namely Decision Tree and Gradient Boosting machine, to predict heart disease. Kumar et al. (28) developed popular RNN model and Reinforcement learning model for COVID-19 prediction. Ngabo et al. (29) proposed several machine learning models and Reinforcement Learning Model for COVID-19 prediction. Iwendi et al. (30) proposed boosted random forest algorithm for COVID-19 disease prediction. Deepa et al. (31) developed an intelligent system based on AI with GDM approaches for healthcare analysis. Dhanamjayulu et al. (32) proposed an image processing technique to identify malnutrition from facial images.

Iwendi et al. (33) proposed a model called N-sanitization, which is used to analyse the unstructured medical datasets for various disease diagnosis. Ahmed et al. (34) used multiple machine-learning models, namely J48, Logistic Regression (LR), and Naïve Bayes (NB). The model of J48 achieved 73.5%, Logistic Regression gained 74.4%, and Naïve Bayes achieved 74.2% with 10-fold cross-validation. Kalra et al. (35) performed a detailed study on diabetic type 1 patients' medical records. Table 1 shows the accuracy details of diabetic positive prediction with respect to existing works.

**Table 1:** Summary of the existing work.

**Author**	**Proposed**	**Accuracy(%)**	**Limitations**
Iyer et al. (6)	J48 Naïve Bayes	74.87 76.96	WEKA tool is used for prediction and prediction accuracy rate is less.
Ahmed (26), Singhania et al. (36)	J48 Logistic Regression model Naïve Bayes classifier	73.5 74.4 74.2	A more extensive study is missed for the data analysis.
Soltani and Jafarian (11)	Probabilistic Neural Network (PNN)	89.56	Type 2 diabetics details only considered for the application development.
Kopitar et al. (22)	Naïve Bayes, Random Forest and KNN	64.47	Diabetic prediction accuracy is less compared with proposed stacking approach.
Ashiquzzaman et al. (7)	DNN, with Dropout	88.41	This method is achieved an 88.41 detection rate. Single approach is used.
Chugh et al. (27)	Decision Tree and Gradient Boosting machine	90.00	The proposed method achieved a 90 accuracy in analyzing diabetes. This paper has focused only on children's data for predicting the diabetics.
Rakshit et al. (12)	Two-class neural network	83.3	This proposed model achieved an 83.3 detection rate of type 2 diabetes. This method has considered the women dataset with their age above 21.
Maniruzzaman et al. (13)	Linear Discriminant Analysis, Quadratic Discriminant Analysis, Naïve Bayes classifier, Gaussian Process modeling	81.97	They accuracy as 81.97, which is less than the proposed method.
Sisodia and Sisodia (9)	Decision Tree SVM Naive Bayes	76.30 65.10 73.82	Diabetic prediction accuracy is less compared to proposed stacking approach.
Rao et al. (21)	Decision Tree with radial function	75.65	Diabetic prediction accuracy is less compared with proposed stacking approach.
Kopitar et al. (22)	XGBOOST	88.4	The obtained accuracy is less and single algorithm XGBOOST is used.
Naveen et al. (23)	SVM, selection Tree, Naive Bayes, Logistic Regression and KNN	75	Several algorithms are used but those algorithms are not combined together for final prediction.
Aishwarya et al. (21), Gadekallu et al. (37), Anup et al. (37)	SVM	95	Single machine learning algorithm is used for prediction.
Kandhasamy and Balamurali (5), Meri et al. (38), Ghazal et al. (39, 40), Hasan et al. (41, 42), Siddiqui et al. (43), Upadhyaya et al. (43), Bakri Hassan et al. (44), Salih Ahmed et al. (45), Ahmed et al. (46), Alsharif et al. (47), Khalifa et al. (48)	J48, KNN, RF, and SVM	73.82	Diabetic prediction accuracy is less compared to proposed stacking approach.

## Method and Techniques

### Ensemble Techniques

In the conventional approach, only one machine algorithm is used for problem-solving. But the single algorithm is not enough for the complex problems. That algorithm may not fit to the input data due to parameter constraints, input data format constraints, and so on. That is the reason that combining more than two machine algorithms, called an “ensemble model,” becomes popular. But the popular question on the ensemble technique is, “How do ensemble models achieve better performance than single approach?” The answer is simple. Just as diversity in nature contributes to more robust biological systems, ensembles of ML models produce stronger results by combining the strengths (and compensating for the weaknesses) of multiple sub models. The proposed system adopts multiple machine learning algorithms (ensemble) to predict the diabetic.

**Figure 1:**
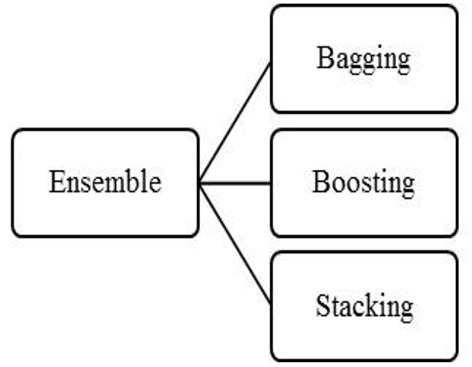
Ensemble techniques.

The Ensemble technique has three categories, bagging, boosting, shown in Figure 1. Each model has its merit and demerits. Among these three, the proposed system has used stacked ensemble modeling for predicting diabetic positive. Table 2 shows the performance analysis of three ensemble models. From Table 2, the stacking is better compared to the other two models in improving the accuracy. In the healthcare system, prediction accuracy is a significant feature to evaluate the system. Since diabetic positive or negative prediction is under the healthcare system, the stacked ensemble approach is used in the proposed model.

**Table 2:** Ensemble techniques comparisons.

**Bagging**	**Boosting**	**Stacked**
Multiple Classifiers are trained parallelly.	Builds the new learner in a sequential way.	Multiple Classifiers are trained parallelly.
The result is obtained by averaging the responses of the N learners.	On each iteration, update the model by weights until the desired result is obtained.	The result is obtained from the second level classifier.
Reduces the variance.	Reduces the bias.	Increases the accuracy.

#### Stacking

Stacking is a two-level classification technique, namely level-0, level-1, or Meta classifier. Unless conventional bagging and boosting, the stacking creates a new training dataset for the final prediction. This approach is entirely different from other multi-classifier algorithms because other multi-classifier approaches use the averaging or voting for the final prediction. But the stacking relays on the predicted probability set, which is generated from all the classifiers. In level 0, more than one algorithm is used. Level 0 works in either a homogeneous or heterogeneous algorithm set. In homogeneous, the same algorithm is used with different parameters, whereas different algorithms are used in heterogeneous. These level-0 algorithms are trained from the original dataset. After the training, the algorithms do not predict the final output. Instead, the probabilities of each class are predicted. Each algorithm predicted the probability of each class and finally generated the predicted probability set. This set will be given as the input to the level-1 algorithm. The level-1 algorithm is trained from the predicted probability set for the final prediction.

**Figure 2:**
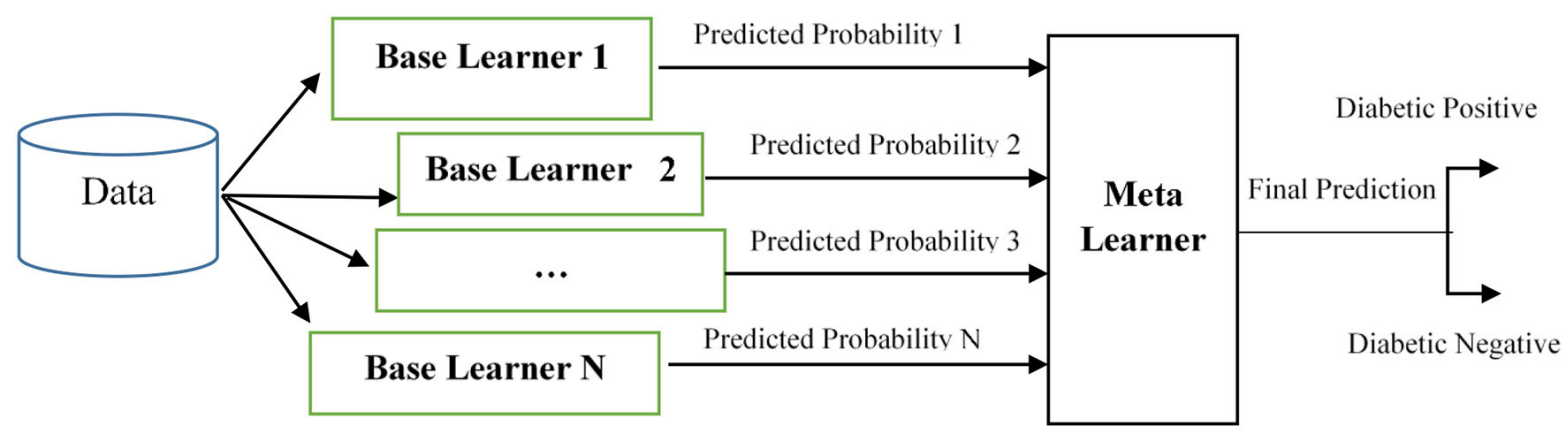
Stacked ensemble model.

The generic model of the proposed stacked ensemble model is shown in Figure 2. In Figure 2, the base learners 1, 2… N are level-0 classifiers, also called as weak learners. The base learners are trained from the dataset to construct the new training set. Meta learner is the level-1 classifier, and it will be trained with the newly created set. After training, the level-1 classifier will predict the test set.

#### Level-0 Classifier

**Figure 3:**
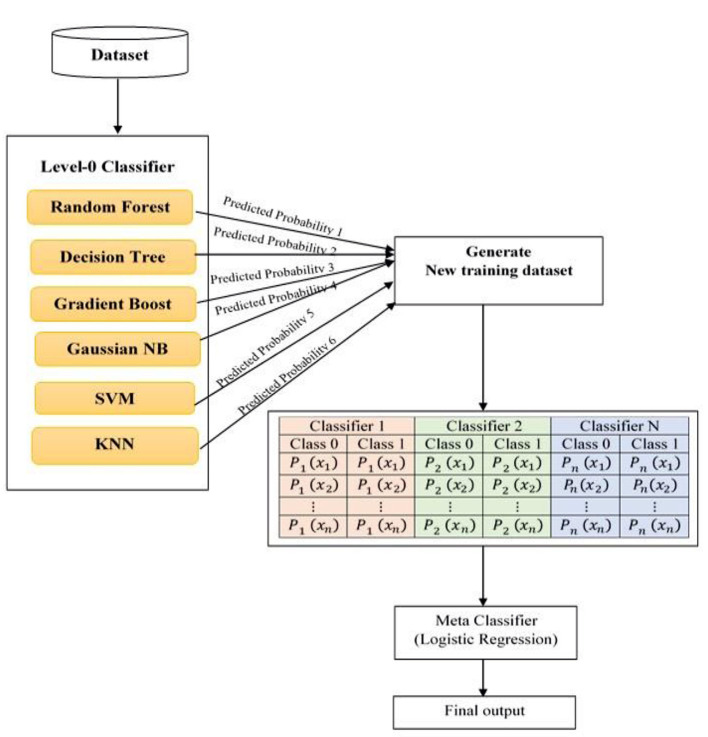
Proposed system: Stacked ensemble model architecture.

Figure 3 depicts the proposed system architecture. The proposed system has selected six different type of machine learning models as level-0 classifiers.

The selected level-0 classifiers are Random Forest, KNN classifier, Decision tree, Gradient Boosting, SVM, and Gaussian Naïve Bayes added as base classifiers. These base classifiers are trained with the original dataset and output a new training dataset for the level-1 classifier.

The advantage of a level-0 classifier in the stacked ensemble technique is two-level classification. In the conventional approach, only one machine algorithm is used for problem-solving. The stacked ensemble learning model is called a multiple classifier system that uses base classifiers to build new training data to classify unknown data. In Figure 3, the level-1 classifier logistic regression is represented as a Meta classifier. The level-0 classifier predicted probability output is given as input to the logistic regression Meta classifier.

#### Level-0 Classifier Input

For the level-0 classifiers, the input is the original dataset. The dataset consists of an input vector set (AttrVec_**1**_, AttrVec_**2**_… AttrVec_**n**_) and the output attribute is **(y**_**1**_**, y**_**2**_**, …, y**_**n**_**)**. The format for the level-0 input is given in Table 3.

**Table 3:** Level-0 Input Set.

**Attribute Set**	**Target output label**
AttrVec_1_ (1^st^ row)	**y**_**1**_
AttrVec_2_ (2^nd^ row)	**y**_**2**_
AttrVec_3_	**y**_**m**_
⋮	⋮
AttrVec_n_ (nth row)	**y**_**2**_

### Proposed System Level-0 Classifiers

#### Random Forest

This is one of the supervised machine learning algorithms. It is used to solve classification and regression problems. The random forest builds the forest from decision trees to solve the problem and improve its performance. The decision tree consists of branches, internal nodes, and leaves. Leaf node represents the final result or class label. Internal nodes are evaluators which decide the branch selection on an attribute (e.g., whether age is eligible to vote or not). The following hyperparameters are used to build the random forest. The number of the estimator is assigned as n_estimators, maximum depth of the tree is assigned as max_depth, minimum number of the split is assigned as min_samples_split, a number of maximum features is assigned as max_features, and the number of a maximum leaf node is assigned as max_leaf_nodes. In addition, n_estimators is a hyperparameter, which indicates the number of decision trees to be generated for the prediction. A higher number of trees is recommended for better prediction, but it may increase the execution time. The default value of n_estimators is 10, and it can be increased up to 500 based on the problem. For the proposed system, the n_estimators is set to 10. That is, every attribute must have min_samples_split samples to divide into two splits. For the proposed method, min_samples_split is set as 2.

Gini index is used to generate the decision tree based on the input dataset. The equation for the Gini index is given below.

(1) $$\begin{array}{r} Gini=1-\overset{c}{\underset{i=1}{\sum }}{\left(pi\right)}^{2} \end{array}$$

C is the total number of classes in the dataset. For our problem, c is assigned as 2. In our Pima dataset, there are a total of eight input attributes and one output class label. The class labels are 1 and 0; 1 indicates the patient has diabetes, and 0 indicates that the patient has not diabetic. *pi* is the probability of selecting the branch among the branches in the ith level for the next level prediction. The proposed system algorithm is shown below.

### K Neighbors Classifier

KNN is one of the supervised machine learning algorithms used for classification and regression problems. KNN finds the relationship between the sets X and Y, where X is the input attribute set and Y is the output data. In the KNN, similar training data points are grouped by capturing the distance between the data points. The lesser distance data points are closer than the broader distance data points. Euclidean distance method is used to compute the distance between the data. The following equation is used to calculate Euclidean distance method.


(2) $$\begin{array}{r} d=\sqrt{\overset{k}{\underset{i=1}{\sum }}{\left(xi-yi\right)}^{2}} \end{array}$$

### Decision Tree Classifier

This is a supervised machine learning model that is used for classification. It is a rule-based approach to solve the classification problem. Decision tree is built from the attribute set by applying the if-else pattern set. To create an if-else pattern set or rule set from the attribute, any one of the Gini index, entropy, or misclassification error methods is followed. The most popular approach is the Gini index. These methods are used to create the decision on the internal node and split the samples for the next level in the tree.

(3) $$\begin{array}{r} Gini=1-\overset{c}{\underset{i=1}{\sum }}{\left(pi\right)}^{2} \end{array}$$

(4) $$\begin{array}{r} Entropy=-\underset{j}{\sum }p_{j}\log_{2}p_{j} \end{array}$$

(5) $$\begin{array}{r} Misclassification\;Error=1-maxp_{j} \end{array}$$

### Gradient Boosting

Gradient boosting is a tree-based machine learning algorithm. Boosting is a method that converts weak learners into strong learners. Initially, a tree is built with the dataset attributes and evaluates the model. In evaluation, the error is calculated by original error minus predicted error. This error is also called a classification error. That is, the rate of misclassification is high. This error is minimized or eliminated by building new trees in the subsequent iterations. The error and the first built tree are considered to build the second tree. The second tree is the improved version of the first model, where the misclassification is reduced while compared to the first model. The new tree is built in every iteration using the previous tree classification error and the previous three. This new tree construction is continued until the error becomes negligible or no changes in the error. The following steps are followed for the classification in the gradient boosting approach.

1. Fit a decision tree to the data: F_1_(x)
2. Fit the following decision tree to the residuals of the previous: h_1_(x) = y–F_1_(x),
3. Add this new tree to our algorithm: F_2_(x) = F_1_(x) + h_1_(x),
4. Fit the next decision tree to the residuals of F_2_: h_2_(x) = y–F_2_(x),
5. Add this new tree to our algorithm: F_3_(x) = F_2_(x)+h_1_(x),
6. Continue this process until the desired output is reached.

The generic formula of GBM is given in Equation 6.


(6) $$\begin{array}{r} f\left(x\right)=\overset{B}{\underset{b=1}{\sum }}f^{b}\left(x\right) \end{array}$$

### Support Vector Machine (SVM)

SVM is a supervised machine learning approach used for both classification and regression problems. SVM is best suited for classification-related problem-solving approaches. In this approach, the data points are placed in the n-dimensional space, where n is the output classes or features. The SVM is well-suited for binary classification than multiclass classification. Equations 7 and 8 are used for computing classification output.

(7) $$\begin{array}{r} w^{T\;}X+b\geq c \end{array}$$

(8) $$\begin{array}{r} w^{T\;}X+b<c \end{array}$$

If the weight matrix and input vector result are higher than c, then the classification output is y1; otherwise, the classification output is y2. Here y1 and y2 are the output class labels.

### Gaussian Naïve Bayes

This is a special type of Naïve Bayes approach and suitable for classification problems. It is a supervised machine learning algorithm. It works under the principle of Bayes theorem. The conditional probability calculation is shown in Equation 9.

(9) $$\begin{array}{r} p\left(Y|x_{1},x_{2},\;\ldots ,\;x_{n}\right)=\frac{p\left(Y\right)p\left(x_{1},x_{2},\;\ldots ,\;x_{n}|Y\right)}{p\left(x_{1},x_{2},\;\ldots ,\;x_{n}\right)} \end{array}$$

#### Level-1 Classifier or Meta Classifier

**Figure 4:**
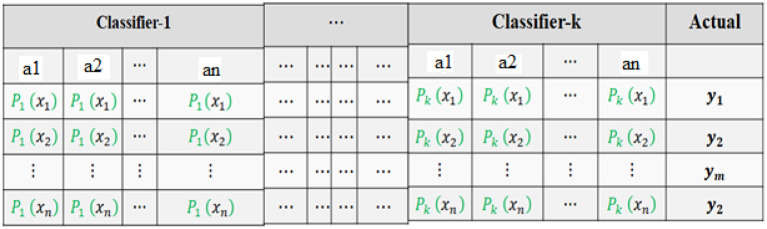
Level-1 classifier input set.

**Figure 5:**
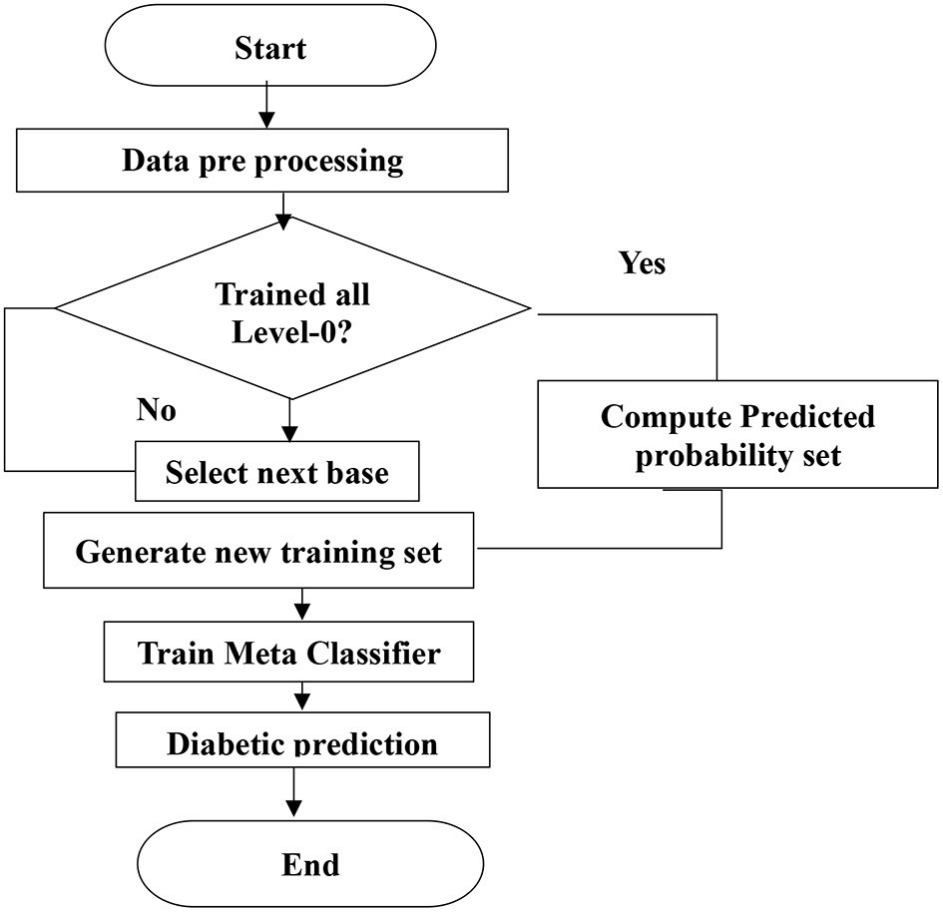
Proposed system flow chart.

Figure 4 shows the level-1 classifier input set. For level 1, the logistic regression model is used for the final prediction; this model is trained with a new training dataset generated in level-0 classifiers. The flow chart for the proposed system is shown in Figure 5.

This dataset consists of set predicted probabilities of each class of each classifier. A row ri is the predicted probabilities base classifiers of each class of ith row of the original dataset. The formula for the final prediction is done by using Equation 10.

(10) $$\begin{array}{r} p=\frac{e^{b0+b1\left(x\right)}}{1+e^{b0+b1\left(x\right)}} \end{array}$$

*b*0, *b*1 are the constants, and x is the input vector. *p* is the final prediction, which is >0.5, then the patient has diabetic positive; otherwise, the patient has diabetic negative.

## Pima Dataset Description

Table 4 shows the Pima dataset attributes description. This dataset consists of 768 rows and nine columns. The last column is the output class, containing 1 and 0; 1 indicates diabetic positive, and 0 indicates diabetic negative.

**Table 4:** Attribute details of Pima Indian Diabetes dataset (PIDD).

**SLO**.	**Feature name with description**	**Feature name (short)**	**Variable type**	**Min val**	**Max val**	**Labeled value**
1	Number of times pregnant- Number of pregnancy	Pregnant	Integer	0	17	Pregnancies
2	Glucose concentration (2-h oral glucose test [mg/dL])	gl	Integer	0	199	Glucose
3	Blood Pressure (Diastolic blood pressure [mm Hg])	bp	Integer	0	122	Blood pressure
4	Skin thickness (Triceps skin fold thickness [mm])	sk	Integer	0	99	Skin thickness
5	Serum Insulin (2-H serum insulin [mu U/mL])	in	Integer	0	846	Insulin
6	BMI (Body Mass Index [kg/m^2^])	bmi	Real	0	67.10	BMI
7	Diabetes Pedigree Function (Diabetes in family history)	dp	Real	0.08	2.42	Diabetes Pedigree Function
8	Age (Age in Years)	age	Integer	21	81	Age
9	Class	Target label	Binary	0 (0-Tested Negative [500])	1 (1-Tested Positive [268])	Target output

**Algorithm 1:** Algorithm Stacked Ensemble.

1.	Input: A training set *D: =* (a_1_, *Y*), (a_2_, *Y*)… (a*_*n*_*, Y)
	Input: A testing set T: =(a_1_, *Y*), (a_2_, *Y*)… (a*_*m*_*, Y)
	where Y: 0 or 1
	Feature set F: {f_1_, f_2_, f_3_, …, f_n_}
2.	Step 1: Assign level-0 classifiers
3.	Number of level-0 learners *l*=6
4.	Step 2: Train the level-0 classifiers using the following
5.	**for** *i* = 1 to *n* do
	**for** *j* = 1 to *l* do
	**assign** (a_j_, *b*_j_) to *l*_i_ **Calculate** predicted probability set ***P***_li_
	**end** for
	**end** for
6.	Step 3: Prepare new training set *(D')*
	*D*' = **(*****P***_l1_, ***P***_l2_, ***P***_l3_, ……, ***P***_l6_, **Y)**,
	**(*****P***_**21**_, ***P***_**22**_, ***P***_**23**_, ……, ***P***_26_, **Y),** **.** **.** **.** **(*****P***_n1_, ***P***_n2_, ***P***_n3_, ……, ***P***_n6_, **Y)**
7.	**Generate Level-0 classifier input set with target output**
	**for** *i* = 1 to *n* do
8.	M_h_ = (a_1_', *Y*), where a_1_' = (***P***_l1_, ***P***_l2_, ***P***_l3_, ……, ***P***_l6_**).** M_h_ – Meta classifier input
9.	**end** for
10.	Step 4: Assign *(D') to* level-1 classifier (*LR*)
11.	Step 5: Train level-1 classifier using *D'*
12.	Step 6: **Prepare** testing set (*D”*) for level-0 classifier without target output
13.	Step 7: **Execute** level-1-classifier(LR) on *D”*
	**for** *i* = 1 to *m* do
	**(*****P***_i1_, ***P***_i2_, ***P***_i3_, ……, ***P***_i6_) **predict Y**.
	**end** for

**Figure 6:**
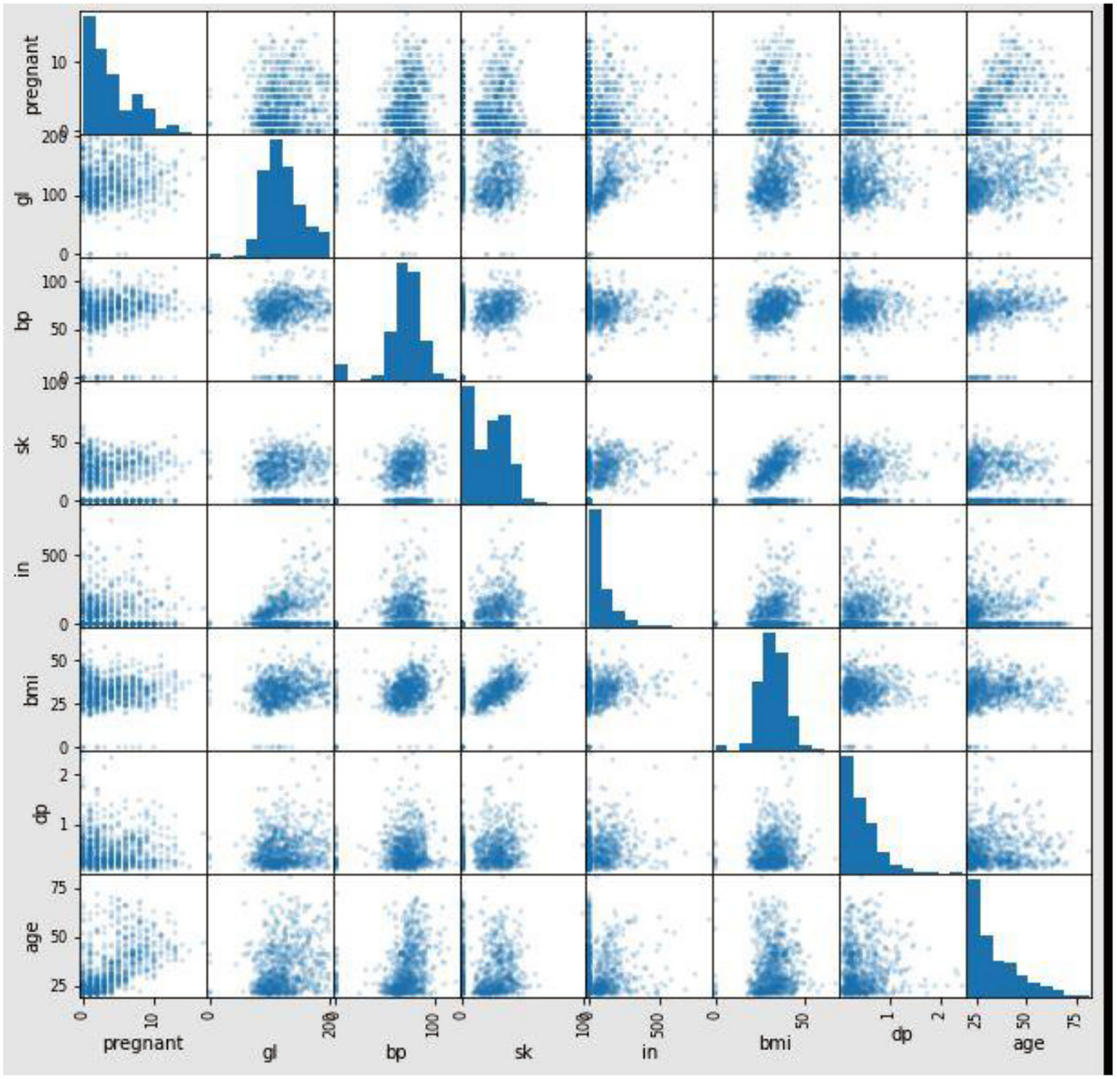
Pearson correlation coefficient of Pima dataset input attributes.

**Figure 7:**
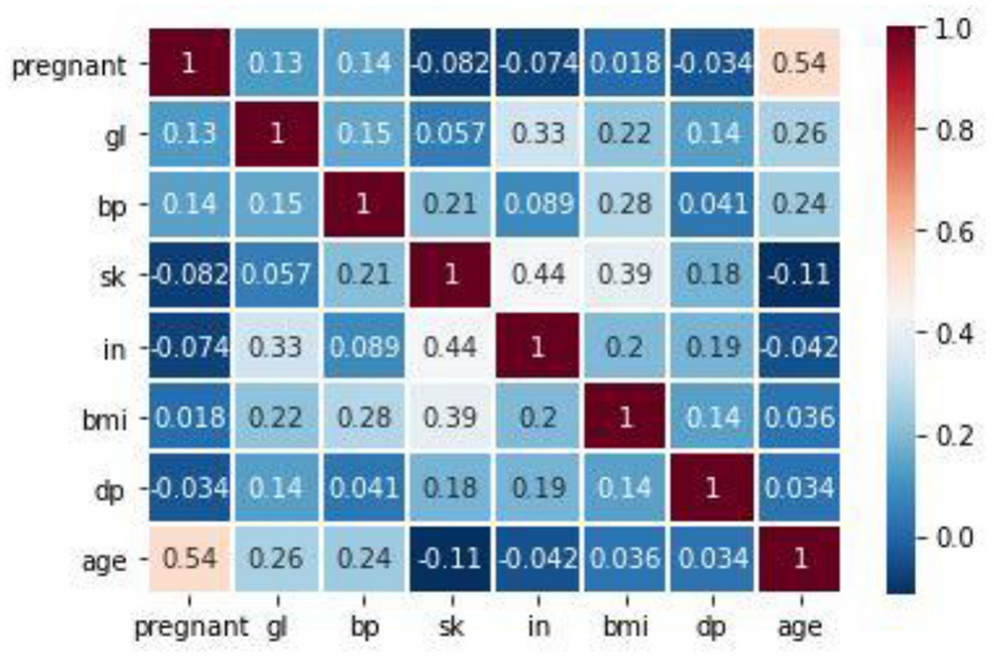
Pearson correlation coefficient result of Pima input attribute set.

Figure 6 shows the correlation between the attributes in the dataset. The proposed method used Pearson's correlation method, which finds the relationship between the variables in the Pima dataset. This correlation says how strong an association or correlation of two attributes. Pearson, correlation coefficient formula, returns a value between −1 and 1. The correlation coefficient between two attributes (X, Y) is 1; then, Y's positive value will also increase for every X positive value increase. If the correlation coefficient between two attributes is negative, then any positive value increase of X, Y's negative value will also decrease. If the correlation coefficient is 0, then there is no relation between X and Y. In Figure 6, for every attribute pair, the Pima dataset correlation is displayed as a scatterplot. In Figure 7, the coefficient value of every two attributes of the Pima dataset is displayed. These two figures depict that most of the attributes in the Pima dataset are independent. The prediction result depends on all the attributes in the dataset.

## Experimental Results and Analysis

**Figure 8:**
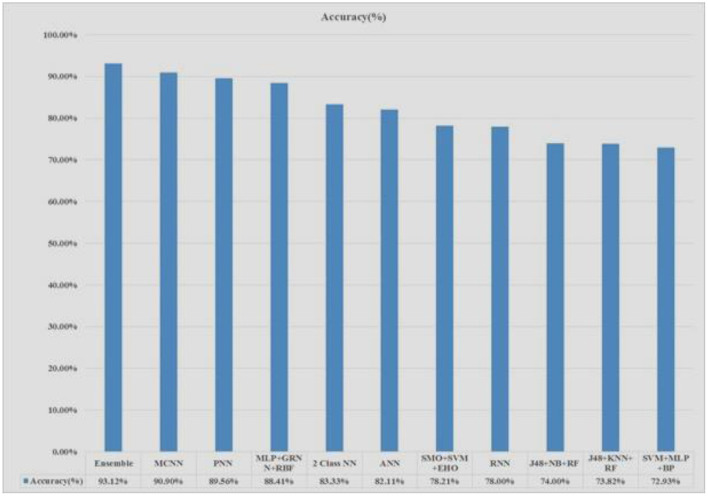
Accuracy chart of various models and proposed model comparison chart.

The Pima Indians Diabetes (PID) Data Set is applied in the experimental purpose. The whole experiment is done in an Intel Core i5- 6200U CPU @ 2.30GHz 4 cores with 4 Gigabytes of DDR4 RAM with the help of python programming language (32, 33, 37). Figure 8 shows that the proposed system stacked ensemble model obtained higher accuracy, around 93%, compared to all other existing models.

True Positive (TP) measures correctly predicted the diabetic patients.
True Negative (TN) measures correctly predicted the non-diabetic patients.
False Negative (FN) measures incorrectly predicted the non-diabetic patients.
False Positive (FP) measures incorrectly predicted the diabetic patients.

(11) $$\begin{array}{r} Precision=\frac{TP}{TP\;+\;FP} \end{array}$$

(12) $$\begin{array}{r} Recall=\frac{TP}{TP\;+\;FN} \end{array}$$

(13) $$\begin{array}{r} F1-Score=2^{*}\frac{Precision^{*}Recall}{Precision\;+\;Recall} \end{array}$$

(14) $$\begin{array}{r} Accuracy=\frac{TP\;+\;TN}{TP\;+\;TN\;+\;FP+FN} \end{array}$$

**Figure 9:**
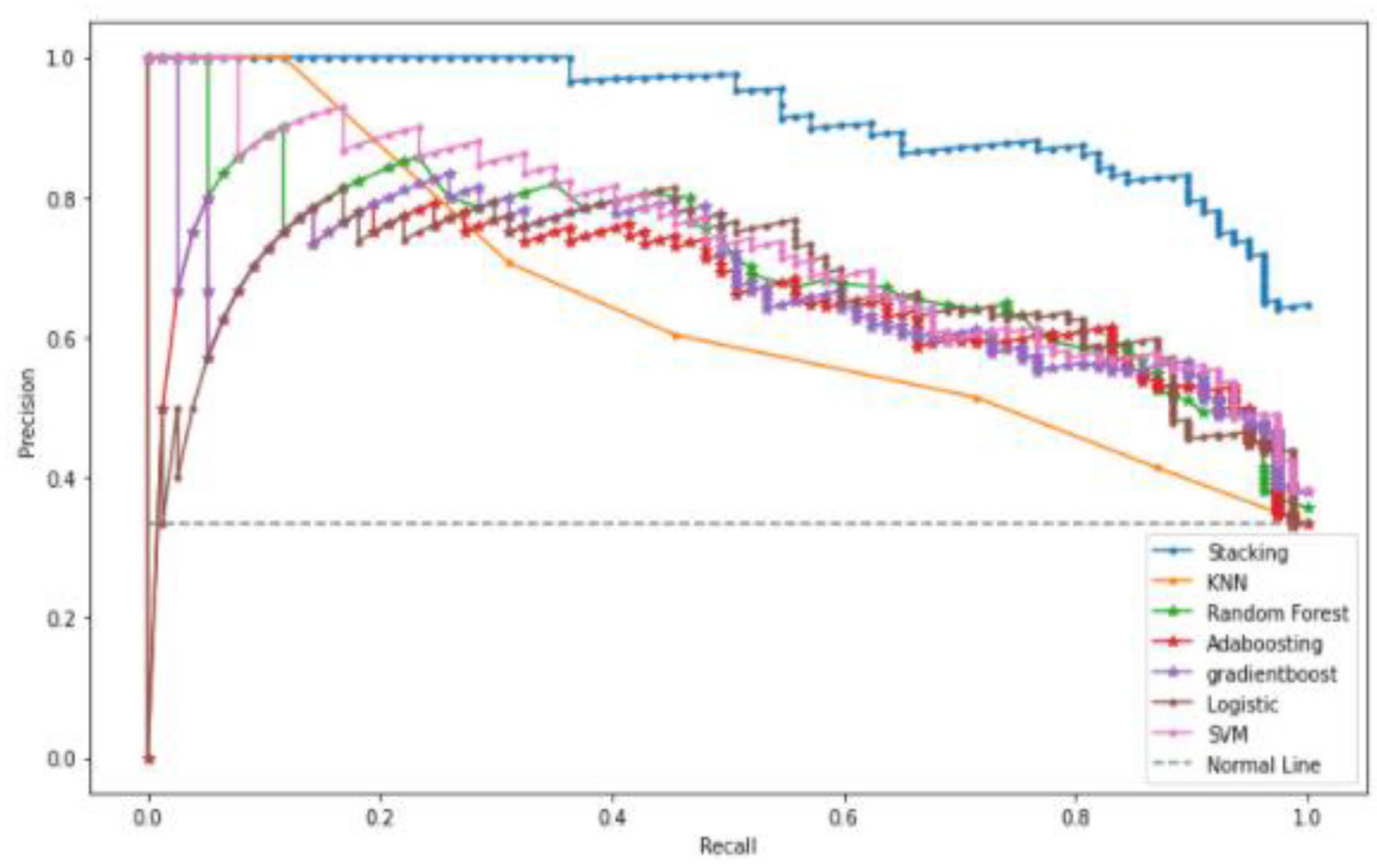
Precision-Recall Curve of the proposed system and various machine learning models.

A Precision-Recall Curve (PRC) is a metric used to compute the quality of the classifier model. The PRC curve is represented in a graph, where X-axis contains recall values Y-axis contains precision values. This curve depicts the compromise between precision and recall. In a graph, the PRC curve occupies a high area, which means that the obtained recall and precision rates are high. High precision leads to a less false positive rate, and high precision leads to a less false-negative rate. Figure 9 shows that the proposed stacked ensemble model curve has occupied a higher area than other machine learning models such as KNN, Random Forest, and Gradient Boosting. The curve values are represented as TP/ (TP+FN) on the Y-axis.

**Figure 10:**
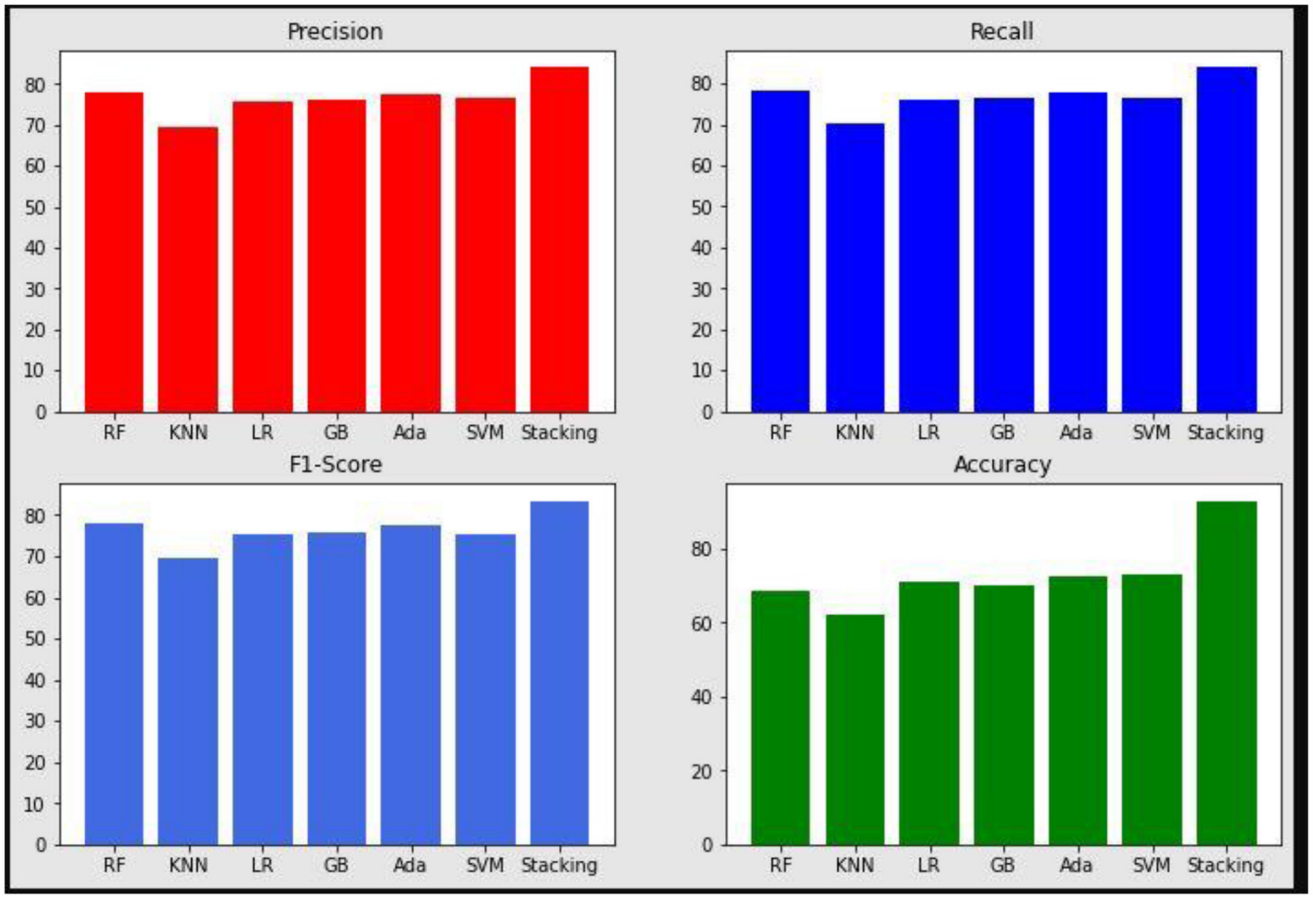
Precision, Recall, F1-Score, and Accuracy results of the proposed system and various machine learning models.

The proposed system is compared with other machine learning models by quality metrics such as precision, recall, accuracy, and F1-score. These values are plotted in Figure 10. The proposed stacked ensemble model obtained higher results compare to all other methods. Table 5 shows the quality metrics results. The proposed method is combination of machine learning algorithms. Generally multiple algorithms for a single problem shows better performance. Each machine learning model has its own strengths and weaknesses. If more than one model is combined, then the weakness may be averaged and strength will be increased for many problems, but not all problems. Thus the ensemble techniques such as bagging, boosting, and stacking are popular. Processing time can be higher than single algorithms. The proposed work is also tested with fewer than 6 machine learning approaches with different combination in ensemble technique and obtained lesser than 93% of accuracy of proposed approach.

**Table 5:** Quality metrics results.

**Methods**	**Precision**	**Recall**	**F1-Score**	**Accuracy**
Random forest	78	78.3	77.8	68.5
KNN	69.3	70.1	69.5	62.4
Logistic regression	75.7	76.2	75.3	71
Gradient boosting	76.1	76.6	75.9	70
Ada boosting	77.9	77.5	77.9	72.7
SVM	76.5	76.6	75.4	73.1
Stacking	84	83.9	83.5	93.1

## Compared With Existing Works

The proposed stacking ensemble model is compared with other machine learning models. The python language is used to implement the proposed model. And the popular machine learning algorithms such as Random Forest, KNN, Logistic Regression, GBM, etc., are implemented and tested with the PIMA dataset. The obtained result is given in Table 5. Compared to the existing models such as Aishwarya et al. (49), Singh et al. (22), and Mamuda and Sathasivam (13), the proposed stacking method has obtained a higher detection rate in detecting the diabetic positive patients.

## Conclusions and Future Works

One of the essential approaches in the medical field is the detection of diseases in the initial stage. Today, diabetes patient increase rates are high irrespective of age across all regions of the world, and there is no medicine (vaccine) to prevent it. The diabetic disease is a big challenge throughout the world, as it affects irrespective of age. Early detection of diabetic positive helps to reduce the medical expenditure, death rate, and risk of patients. As long as the early prediction on this disease is not famous, the proposed system initiated the prediction of diabetic positive. Experiments are carried out on the Pima Indians Diabetes Database (PIDD). A stacked ensemble model has been adopted in the proposed work and obtained 93% accuracy for a highly categorical dataset.

The existing models in the diabetic prediction used a single algorithm. But the single algorithm will not be suitable for the unstructured and large datasets. Thus, the proposed system has adopted multiple machine learning models called stacked ensemble models. The proposed prediction model has predicted diabetic patients accurately about 93% of the time. In the future, the designed system with the used stacked ensemble method can predict other diseases. The work can be extended and improved for the automation of diabetes analysis, including machine learning and deep learning algorithms.

## Data Availability Statement

The original contributions presented in the study are included in the article/supplementary materials, further inquiries can be directed to the corresponding author.

## Author Contributions

SR, SM, MH, SA, SA-K, and RS: concept, methodology, validation, analysis, supervision, writing, drafting, review and editing, funding, software, and supervision. All authors contributed to the article and approved the submitted version.

## Funding

This research has been supported by the Universiti Kebangsaan Malaysia under research grant GP-2021-K023308, and Taif University Researchers Support, Taif University, Taif, Saudi Arabia.

## Conflict of Interest

The authors declare that the research was conducted in the absence of any commercial or financial relationships that could be construed as a potential conflict of interest. The reviewer SM declared a shared affiliation with the authors to the handling editor at the time of review.

## Publisher's Note

All claims expressed in this article are solely those of the authors and do not necessarily represent those of their affiliated organizations, or those of the publisher, the editors and the reviewers. Any product that may be evaluated in this article, or claim that may be made by its manufacturer, is not guaranteed or endorsed by the publisher.
